# Social media use and life satisfaction among elderly migrants: dual mediation of social connection and place attachment

**DOI:** 10.3389/fpsyg.2026.1812028

**Published:** 2026-08-27

**Authors:** Feng Yuan

**Affiliations:** Yangtze Normal University, Chongqing, China

**Keywords:** elderly migrants, life satisfaction, place attachment, social connection, social media use

## Abstract

**Background:**

“Elderly migrants” constitute an important segment of the aging mobile population. Due to their mobility, their mental health and psychological needs require greater attention. Life satisfaction serves as a key indicator of their mental health. Therefore, understanding the life satisfaction of elderly migrants is a critical and practical research topic. However, the impact of social media use on improving their life satisfaction has not been empirically verified.

**Methods:**

This study employs a structural equation model to propose research hypotheses and a theoretical framework, followed by empirical testing. Specifically, the study first conducts a literature review to propose a structural equation model using social connection and place attachment as theoretical perspectives and mediators. This model explores whether the social media use of elderly migrants can enhance their life satisfaction by improving their social connection and sense of place attachment, thus addressing their psychological needs. A sampling method was then used to select participants for the study, and a survey was conducted, yielding 328 valid responses. Finally, empirical methods were employed to verify the hypotheses.

**Results:**

(1) Social media use among elderly migrants positively impacts social connection and place attachment. (2) Social media use among elderly migrants indirectly and positively influences life satisfaction through the mediating roles of social connection and place attachment, thereby meeting psychological needs. (3) Social media use among elderly migrants does not directly and positively affect life satisfaction.

**Conclusion:**

Social media use has a definitive impact on the life satisfaction of elderly migrants. Therefore, it is recommended that elderly migrants learn to use various social media functions to enhance their convenience. Children should provide digital support to their accompanying parents, while social media platforms should develop age-friendly functions and products. Additionally, governments, communities, and social organizations should facilitate online and offline interactions to help elderly migrants establish social connections and a sense of place attachment, thereby improving their life satisfaction, addressing their psychological needs, and promoting mental health.

## Introduction

Population aging has become one of the most prominent features of Chinese society. According to the 2023 National Report on Aging Development, the total elderly population in China is projected to reach approximately 420 million by around 2035, accounting for more than 30% of the population and signaling a transition into a heavily aged society (Ministry of Civil Affairs, 2024). Corresponding to the deepening aging process, the 2023 Report on the Development of China’s Migrant Population indicates that the scale of elderly migrants is steadily increasing, with younger seniors being the majority. The three main reasons for elderly migration are caregiving for younger generations, retirement living, and employment (National Health Commission, 2023).

Under the dual forces of population aging and mobility, elderly migrants have formed a substantial social group colloquially referred to as the “elderly drifters.” Regardless of their reasons for migrating, they contribute to urban development in new ways. However, in the unfamiliar urban environment, leaving behind the familiar social networks of their hometowns, elderly migrants inevitably face challenges in living, recreation, and socializing. These issues affect their sense of security, belonging, and life satisfaction, potentially leading to unmet psychological needs and impacting their mental health.

The concept of “elderly migrants” has yet to achieve a unified definition in academic circles, with varying classifications and criteria. Many scholars define elderly migrants as older adults who leave their hometowns to live in unfamiliar cities with their children to provide care for their children and grandchildren, with intergenerational caregiving being their primary task in their new residence (Liu, 2012). Yang et al. (2018) identified three main reasons for elderly migration: caregiving for younger generations, retirement, and employment or business. From the perspective of public mobility, Lu and Zheng (2016) categorized elderly migrants based on two dimensions: whether their children have obtained urban household registration and whether they are providing care for grandchildren. They divided elderly migrants into dual-migrant, nanny-type, laborer-type, and dependent categories (Lu and Zheng, 2016). This study includes all mobility-related elderly groups regardless of household registration type.

The age range of elderly migrants is also debated. The United Nations World Health Organization defines older adults as those aged 60 and above. However, Wu (2014) argues that “elderly” in the context of elderly migrants refers specifically to grandparents in the traditional familial sense. Similarly, Xu and Hua (2018) includes both pre-elderly (aged 50 and above) and elderly individuals in the elderly migrant group. Considering China’s classification of migrant workers, which also caps the highest age category at 50 years and above (Bank of China Research Institute, 2021), this study defines elderly migrants as individuals aged 50 and older.

In recent years, as China’s aging society has deepened, scholars have increasingly focused on the life satisfaction of older adults. Existing studies have demonstrated that internet use has a significant positive effect on life satisfaction among the elderly (Du and Wang, 2020). According to the 54th Statistical Report on the Development of China’s Internet, as of June 2024, individuals aged 60 and above accounted for 14.3% of internet users (China Internet Information Center, 2024), including elderly drifters. By using specific social media platforms, these individuals can establish real-time connections with familiar social networks in the virtual space and access social information anytime, anywhere via mobile devices.

However, does social media use help elderly drifters form social connections and develop a sense of attachment to their new place of residence? Does it have a significant positive effect on their life satisfaction?

In summary, this study attempts to construct an integrated model using social connection and place attachment as mediating variables to explain and predict the relationship between social media use and life satisfaction among elderly drifters. The structure of this paper is as follows: first, it reviews and organizes existing literature on the relationships among social media use, social connection, place attachment, and life satisfaction. Next, it proposes corresponding hypotheses and establishes a research model, followed by explanations of the research design and methods, data analysis, and results. Finally, the study concludes with key findings and relevant recommendations.

## Literature review and conceptual model

### Theoretical foundation: uses and gratifications theory

Uses and gratifications (U&G) theory views media users as active participants who deliberately select and use media to satisfy their needs, rather than passively receiving media content (Katz et al., 1974). From this perspective, media use is goal-oriented behavior through which individuals seek various forms of gratification. Applied to social media, U&G theory suggests that users actively engage with social media platforms to satisfy a variety of social and informational needs (Quan-Haase and Young, 2010). Social media facilitates the maintenance and development of interpersonal relationships, enhances social interaction, and provides convenient access to information and knowledge about others (Whiting and Williams, 2013). For groups experiencing major life transitions or relocation, such as elderly migrants, these gratifications are especially salient.

Against this theoretical backdrop, the social media use of elderly migrants can be understood as a way to manage their integration and connections with both urban and hometown environments, yet systematic research on this topic remains limited. Cheng et al. (2026) found that Social media use can serve as an important “digital bridge,” which not only helps the elderly migrant maintain their original social relationships and provide emotional support, but also may expand their social connection and integration channels in the new environment through regional or interest-based participation. Ugalde et al. (2023) argue that social media usage improves the quality of life and health of the elderly.

Taken together, these theoretical and empirical insights suggest that elderly migrants’ social media use can be viewed as an active strategy to cope with the social and environmental challenges of migration. In line with U&G, they use social media not only to preserve existing ties with their hometown networks, but also to build new connections and enhance their adaptation to the current living environment. Accordingly, this study examines how social media use may influence elderly migrants’ social connection, place attachment, and life satisfaction, and tests these relationships in the structural model proposed in the following sections.

### Social connection and social media use

Social connection refers to a sense of belonging and intimacy with others and social collectives (Lee and Robbins, 1995). It acts as a universal social lens through which people perceive and interact with the world (Li et al., 2008). Research by Williams and Galliher (2006) confirmed that social connection reduces levels of anxiety, shame, and depression, serving as a protective factor against emotional problems. Conversely, individuals with low social connection often feel disconnected, leading to prolonged states of loneliness and low self-esteem (Lee et al., 2002). In this study, social connection is defined as the sense of belonging and intimacy elderly migrants experience with interpersonal relationships and the social environment in their new residence.

Existing research on social connection often considers it a dimension of social capital. Chang and Chuang (2011) argued that social connection directly influences knowledge-sharing behavior and indirectly affects it by enhancing an individual’s sense of belonging and satisfaction within knowledge communities. Scholars have also explored the relationship between internet use, social connection, and mental health among older adults (Hong et al., 2015). Zhang and Du (2026) argue that digital social connection has become the communicative foundation for the construction of an individualized society.

Wang (2019) suggests that the development and popularization of smartphones and mobile social media have successfully integrated elderly migrants into the digital network. Xu and Hua (2018) found that advanced communication tools help elderly migrants maintain contact with relatives in their hometowns, alleviating feelings of alienation and loneliness in urban settings. Building on this evidence, the present study argues that social media use is an important means for elderly migrants to strengthen social connection by maintaining existing ties and fostering new ones across geographical distance. Therefore, this study proposes the following hypothesis:

*H1:* Social media use by elderly migrants has a significant positive effect on their social connection.

### Life satisfaction and social media use

Life satisfaction refers to an individual’s overall cognitive evaluation of their life conditions over most of the time or a specific period, based on their chosen standards (Shin and Johnson, 1978). Diener and Diener (1995) describe life satisfaction as a critical indicator of national well-being and a subjective assessment of overall life quality. Yan and Liu (2025) argue that the life satisfaction of the elderly is an important indicator for measuring their quality of life. Enhancing their life satisfaction is of great significance for improving the social well-being of the elderly.

Research on life satisfaction among older adults began in the 1960s in the United States, while similar studies in China started in the mid-1980s (Zhang and Xu, 2021). Around 2000, as China entered an aging society, domestic scholars increasingly focused on life satisfaction among older adults. Xiao et al. (2018) found that loneliness and self-rated health mediate the relationship between social support and life satisfaction among rural older adults. Li et al. (2008) highlighted that marital status significantly affects depressive emotions among non-empty-nest elderly individuals. In particular, Zhang et al. (2024) argues that active and passive social media use have different effects on life satisfaction among older adults, indicating that the ways in which older people engage with social media are closely related to their overall life satisfaction and providing a basis for further examining the role of mobile social media use.

Studies on elderly migrants remain scarce. Jin and Liu (2017) found that increased caregiving intensity for grandchildren significantly enhances life satisfaction among older adults. However, greater caregiving intensity reduces life satisfaction for older women while increasing it for older men (Jin and Liu, 2017).

However, most existing studies have focused on older adults in general, and have seldom addressed elderly migrants as a specific group. In particular, empirical evidence on the relationship between mobile social media use and life satisfaction among elderly migrants remains limited. Therefore, this study proposes Hypothesis 2:

*H2:* Social media use by elderly migrants has a significant positive effect on their life satisfaction.

### Place attachment and social media use

Place attachment refers to the social bonding between individuals and a specific place (Nelli and Tara, 2013), where identifying with a place is a process of self-belonging. Hernández et al. (2007) define place attachment as a positive emotional connection between individuals and a specific place, characterized by a tendency to maintain intimacy with that place. Williams et al. (1992) divide place attachment into two dimensions: place dependence and place identity. The mainstream view considers place attachment as an organic combination of place dependence and place identity, which is the dimensional framework adopted in this study.

Place attachment, rooted in environmental psychology, examines how individuals develop feelings toward significant places in their lives. This is a core focus of place attachment research (Hernández et al., 2007). Recent studies have found that platforms like TikTok can foster a sense of place, generate new meanings for places, and act as a new way to build social cohesion (Huang, 2020). Wang et al. (2020) discovered that anti-pandemic music videos shared through social media had a significant positive impact on tourists’ place attachment. Zhuang (2024) found that the use of social media can help elderly accompanying migrants aggregate and expand their offline relationships, thereby forming place attachment. These findings indicate that social media platforms can function as important channels for constructing and strengthening individuals’ attachment to specific places.

The literature reveals limited research on place attachment among elderly migrants in new residences. However, studies suggest that migrants aspire to identify with their new residences, and strong place attachment can compensate for losses incurred by migration, enhancing psychological and cultural adaptation (Greif, 2006). Based on this discussion, this study proposes the following hypothesis:

*H3:* Social media use by elderly migrants has a significant positive effect on their place attachment.

### Social connection, place attachment, and life satisfaction

Social connection links individuals and provides social support. For elderly migrants, previously established offline social connection networks may be disrupted due to spatial and temporal migration. Nimrod (2010) found that older adults effectively form online social connections through social media and new media technologies. The internet offers social connection support that transcends temporal and geographical boundaries. Wright (2000) discovered that older adults who actively use the internet experience less stress and higher life satisfaction than non-users. Lai (2018)’s empirical analysis revealed that social connection significantly predicts life satisfaction among senior learners. Zhang et al. (2024) found active social media use could indirectly aflect life satisfaction among olderadults through the mediation of online social connectednes and social capital.

Existing studies have also established a significant relationship between place attachment and well-being (Wiles et al., 2009). However, place attachment takes a long time to develop into a stable variable. For migrants, compared to local residents, place attachment and its relationship to life satisfaction are particularly significant, suggesting that migrants’ place attachment can positively predict their life satisfaction. Based on the literature review and discussion, this study proposes the following hypotheses:

*H4:* Social connection has a significant positive effect on life satisfaction among elderly migrants.

*H5:* Place attachment has a significant positive effect on life satisfaction among elderly migrants.

*H6:* Social connection mediates the relationship between social media use and life satisfaction among elderly migrants.

*H7:* Place attachment mediates the relationship between social media use and life satisfaction among elderly migrants.

**Figure 1:**
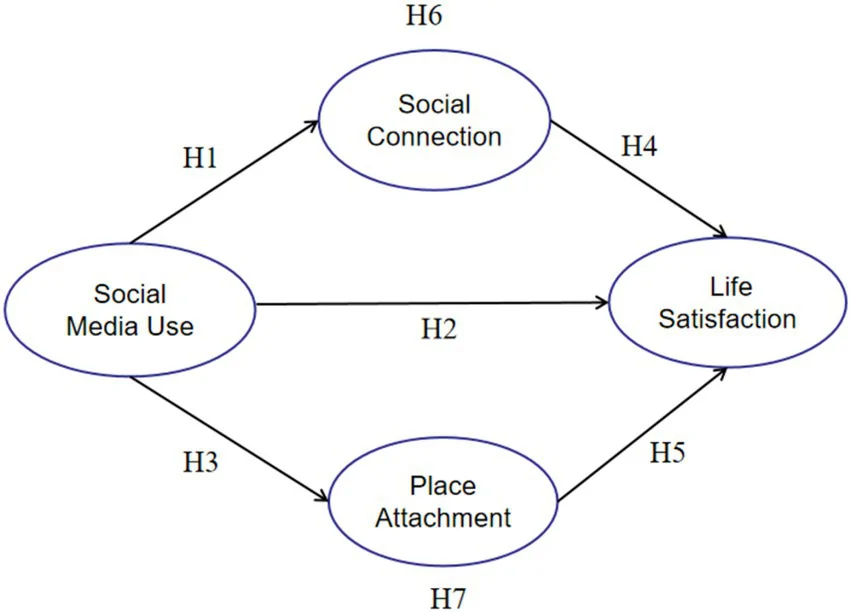
Research model and hypotheses.

In conclusion, this study presents the research model shown in Figure 1, which uses social connection and place attachment as mediating variables to construct an integrated model explaining and predicting the relationship between social media use and life satisfaction among elderly migrants, as well as its influencing factors.

Most extant studies have examined the pairwise relationships among social media usage, social connection, local attachment, and life satisfaction in isolation. They have not yet integrated these four variables into a unified framework, which makes it challenging to systematically uncover multiple interaction pathways. Meanwhile, the “elderly migrants” possess unique mobility characteristics of “leaving their hometowns—relocating—rebuilding their lives.” Their local attachment is highly reliant on social connections. Nevertheless, most existing theories are grounded in local residents or general immigrant groups, lacking explanations and predictive models for the digital adaptation and local sense construction mechanisms of this group.

This study, for the first time, incorporates four core variables into a unified framework, constructs an integrated model with social connection and local attachment as dual mediators, systematically reveals the formation path of life satisfaction among the “elderly migrant workers,” and proves that the use of social media indirectly plays a role through two parallel paths: “enhancing social connection” and “strengthening local attachment”.

In addition, extending the theory of local attachment from long-term residents to the group of elderly migrants provides a theoretical basis for understanding how the elderly migrants cope with the transitional predicament, and enriches the cross-disciplinary achievements of environmental psychology, immigrant adaptation and geriatric transmission research.

## Research methods

### Survey participants and sampling method

This study explores the relationship between social media use and life satisfaction among elderly migrants. According to the 2023 Report on the Development of China’s Migrant Population released by the National Health and Family Planning Commission, the total number of elderly migrants living away from their hometowns has exceeded 20 million, accounting for approximately 10% of the elderly population, which constitutes the population for this survey. Based on the literature analysis of the definition of elderly migrants, this study defines elderly migrants as individuals aged 50 and above, including those engaged in intergenerational caregiving, work, or retirement, as the research sample. A questionnaire survey was conducted anonymously through Wenjuanxing (an online survey platform) from August 15 to 27, 2021. The survey link was initially distributed via WeChat Moments, WeChat groups, and QQ groups, inviting eligible relatives and friends to complete the questionnaire and help forward it. Additionally, 60 undergraduate students from Yangtze Normal University were organized into survey teams, each seeking 3–4 respondents in their respective hometowns and assisting them in completing the questionnaire on their mobile devices. Moreover, face-to-face interviews were conducted at a residential community and a community health service center in Fuling District, Chongqing, resulting in the collection of 24 responses (Du, 2019). A total of 331 questionnaires were collected during this survey, covering 21 provinces, cities, and autonomous regions, including Chongqing, Shanghai, Zhejiang, Guangdong, and Guangxi. After excluding invalid responses with uniform answers and those from participants who did not use social media, 328 valid questionnaires were retained, yielding an effective rate of 99.1%. This sample size meets the recommended range for structural equation modeling (SEM) of 250–500, as suggested by Schumacker and Lomax (Schumacker and Lomax, 2004). As a result, the final sample consisted primarily of active social media users. Therefore, the findings of this study should be interpreted within the context of elderly migrants who use social media and may not be fully generalizable to the broader elderly migrant population.

### Questionnaire design

The questionnaire is divided into two parts: (1) The relationship between social media use and life satisfaction among elderly migrants, which includes four dimensions: social media use, social connection, place attachment, and life satisfaction. (2) Personal demographic information about elderly migrants, including gender, age, and pre-retirement occupation. The questionnaire adopts a seven-point Likert scale, where “1” indicates “strongly disagree,” and “7” indicates “strongly agree.” The questionnaire design was reviewed and refined by industry experts and scholars. The final questionnaire includes four dimensions, 22 measurement indicators, and references from the literature, as summarized in Table 1.

**Table 1:** Summary of dimension measurement methods.

Variable	Indicator	Indicator description	Literature reference
Social media use	Q1	I share many of my daily activities via social media.	Jenkins-Guarnieri et al. (2013) and Wei and Chen (2015)
	Q2	I like, share, and comment on social media.	
	Q3	I follow updates from old friends on social media.	
	Q4	I follow news events on social media.	
	Q5	I join group chats via social media.	
		I shop using social media.	
Social connection	Q1	Using social media gives me a sense of belonging.	Lai (2018)
	Q2	Using social media makes me feel emotionally supported.	
	Q3	Using social media makes me feel more connected to society.	
	Q4	Using social media reduces my feelings of loneliness.	
	Q5	Using social media reduces my anxiety.	
Place attachment	Q1	I have found the life I want to live here.	Zhang and Song (2010) and Qiu Fan et al. (2014)
	Q2	Living here gives me a sense of security.	
	Q3	I have many fond memories of living here.	
	Q4	Compared to other places, I prefer living here.	
	Q5	I feel a sense of belonging and being part of this place.	
	Q6	I feel at home here, and leaving would make me sad.	
Life satisfaction	Q1	I am satisfied with my current living environment.	Lai (2018) and Diener et al. (1985)
	Q2	I am satisfied with my current interpersonal relationships.	
	Q3	I am satisfied with my current financial situation.	
	Q4	I have achieved what I want most in life.	
	Q5	If I could choose again, I would not change anything.	

Data source: organized by this study.

All measurement items were administered in Chinese and adapted from previously validated Chinese-language scales. Therefore, no cross-language translation procedure was required during data collection. For manuscript preparation, the English descriptions of the items were translated and reviewed by the authors to ensure consistency with the original meanings.

Although some items in the Social Media Use scale involve interaction-related behaviors (e.g., following updates from old friends and joining group chats), these items were designed to capture patterns and frequency of social media use rather than respondents’ perceived quality of social relationships. In contrast, Social Connection refers to individuals’ subjective sense of belonging, interpersonal connectedness, and social support. Therefore, the two constructs remain conceptually distinct.

In addition to communication and social interaction functions, contemporary social media platforms increasingly serve as channels for information acquisition, entertainment, and online consumption. For elderly migrants, platforms such as WeChat are frequently used not only to maintain social relationships but also to obtain product information and conduct online purchases. Therefore, the item “I shop using social media” was retained as one indicator of social media use in this study.

### Method selection

Structural Equation Modeling (SEM) is a confirmatory multivariate statistical analysis method often used to test complex hypothetical relationships among variables. The main reasons for choosing SEM in this study are as follows: (1) SEM integrates and improves other analytical methods, allowing for measurement errors in both independent and dependent variables. It combines various indicators to evaluate the goodness of fit of theoretical models, thereby improving the accuracy of research results. (2) This study follows a process of proposing research hypotheses and theoretical models, then validating them empirically, aligning with the confirmatory analysis principles of SEM. (3) The effective sample size for this study is 328, which meets the large sample requirement of SEM (above 300), making this method appropriate and reasonable.

### Common method bias consideration

Given that all variables in this study were measured using a self-reported questionnaire administered at a single point in time, common method bias (CMB) may pose a potential threat to the validity of the findings. To mitigate this risk, several procedural remedies were implemented during the data collection process. First, the survey was conducted anonymously to reduce social desirability bias and respondents’ evaluation apprehension (Podsakoff et al., 2003). Second, participation was entirely voluntary, and respondents were assured that there were no correct or incorrect answers, thereby minimizing response bias. Third, all measurement items were adapted from well-established scales that have demonstrated satisfactory reliability and validity in prior research. Despite these procedural safeguards, the potential influence of common method bias cannot be entirely ruled out.

## Data analysis and results

### Frequency table

The frequency table indicates that the basic data surveyed in this study includes gender, age, reasons for migration, types of migration, and frequency of social media use. Among all respondents, females accounted for a higher proportion, with 211 individuals (64.33%). Two-thirds of the respondents were aged between 50 and 60 years, totaling 218 individuals (66.46%). More than 40% of respondents indicated caregiving for grandchildren as their reason for migration, with 141 individuals (42.99%). The number of intra-provincial migrants and inter-provincial migrants exceeded one-third, at 120 individuals (36.59%) and 115 individuals (35.06%), respectively. Regarding the frequency of social media use, nearly 70% of respondents used social media daily, with the largest group of 221 individuals (67.38%). The demographic results of the respondents are shown in Table 2.

**Table 2:** Frequency table.

Variable	Value label	Frequency	Valid percent
Gender	Male	117	35.67
	Female	211	64.33
		328	100.0
Age	50–60 years	218	66.46
	61–70 years	84	25.61
	71–80 years	18	5.49
	Over 80 years	8	2.44
		328	100.0
Reason for migration	Care giving for grandchildren	141	42.99
	Employment	97	29.57
	Retirement	50	15.24
	Other	40	12.20
		328	100.0
Type of migration	Inter-provincial	115	35.06
	Intra-provincial	120	36.59
	Local	93	28.35
		328	100.0
Frequency of social media use	Never	32	9.76
	Two to three times per month	24	7.32
	Two to three times per week	51	15.55
	Daily	221	67.38
		328	100.0

### Descriptive statistics

The total number of valid questionnaires was 328. The mean values ranged from 3.83 to 5.37, and the standard deviations were between 1.26 and 1.99. Skewness and kurtosis values indicated compliance with normal distribution. As shown in the Table 3, the item “SAT01: I am satisfied with my current living environment” had the highest mean value of 5.37, indicating the highest level of agreement among respondents. Conversely, “SOME06: I frequently shop using social media platforms such as WeChat and TikTok” had the lowest mean value of 3.83, reflecting lower levels of agreement.

**Table 3:** Descriptive statistics (*N* = 328).

Variable	Mean	Std. dev.	Kurtosis	Skewness
SOME0l	4.38	1.91	−1.12	−0.30
SOME02	4.63	1.83	−0.81	−0.53
SOME03	5.26	1.67	0.34	−1.06
SOME04	5.11	1.67	0.14	−0.97
SOME05	4.30	1.95	−1.17	−0.18
SOME06	3.83	1.99	−1.26	0.03
SOCO0l	4.96	1.56	−0.18	−0.76
SOCO02	4.70	1.66	−0.54	−0.55
SOCO03	5.24	1.40	0.42	−0.91
SOCO04	4.58	1.63	−0.67	−0.34
SOCO05	4.71	1.60	−0.57	−0.45
PLAT01	4.61	1.55	−0.61	−0.37
PLAT02	4.44	1.67	−0.85	−0.23
PLAT03	4.70	1.51	−0.41	−0.44
PLAT04	4.15	1.71	−0.96	−0.02
PLAT05	4.44	1.60	−0.88	−0.18
PLAT06	3.98	1.74	−0.95	0.08
SAT01	5.37	1.26	1.66	−1.08
SAT02	5.22	1.33	0.90	−0.88
SAT03	5.03	1.43	0.51	−0.86
SAT04	4.77	1.60	−0.58	−0.51
SAT05	4.46	1.74	−0.94	−0.23

SOME: Social Media Use; SAT: Life Satisfaction; SOCO: Social Connection; PLAT: Place Attachment.

## Model analysis and hypothesis testing

### Reliability and validity testing

As shown in the Table 4, standardized factor loadings range from 0.651 to 0.885. According to Chin’s viewpoint, standardized factor loadings greater than 0.60 are acceptable, and values greater than 0.70 are ideal (Chin, 1998). Thus, all items fall within the recommended range, indicating adequate item reliability for each question. The composite reliability of the constructs ranges from 0.887 to 0.938, all exceeding 0.7, which meets the standards suggested by Nunnally and Bernstein (1994) and Fornell and Larcker (1981), demonstrating good internal consistency for each construct. Finally, the average variance extracted (AVE) ranges from 0.578 to 0.72, all above 0.5, meeting the criteria recommended by Hair et al. (1998) and Fornell and Larcker (1981), indicating good convergent validity for each construct.

**Table 4:** Results of measurement model analysis.

Construct	Item	Item reliability		Construct reliability	Convergence validity
				Std.	SMC	CR	AVE
SOME	SOME01	0.806	0.650	0.891	0.578
	SOME02	0.864	0.746		
	SOME03	0.730	0.533		
	SOME04	0.736	0.542		
	SOME05	0.759	0.576		
	SOME06	0.651	0.424		
SOCO	SOCO01	0.846	0.716	0.928	0.720
	SOCO02	0.866	0.750		
	SOCO03	0.778	0.605		
	SOCO04	0.863	0.745		
	SOCO05	0.885	0.783		
PLAT	PLAT01	0.835	0.697	0.938	0.717
	PLAT02	0.882	0.778		
	PLAT03	0.802	0.643		
	PLAT04	0.856	0.733		
	PLAT05	0.881	0.776		
	PLAT06	0.821	0.674		
SAT	SAT01	0.797	0.635	0.887	0.611
	SAT02	0.787	0.619		
	SAT03	0.782	0.612		
	SAT04	0.795	0.632		
	SAT05	0.746	0.557		

Unstd.: Unstandardized factor loadings; Std: Standardized factor loadings; SMC: Square Multiple Correlations; CR: Composite Reliability; AVE: Average Variance Extracted.

### Discriminant validity

This study uses the AVE method to test discriminant validity. Fornell and Larcker (1981) suggest that discriminant validity should consider both convergent validity and construct correlations. They recommend that the square root of the AVE for each construct should be greater than the correlation coefficients between constructs. If this condition is met, it indicates that the model has discriminant validity. As shown in the Table 5, the square root of the AVE for each construct on the diagonal is greater than the off-diagonal correlation coefficients, demonstrating that the model possesses discriminant validity.

**Table 5:** Discriminant validity of measurement model.

Construct	AVE	SOME	SOCO	PLAT	SAT
SOME	0.578	**0.760**			
SOCO	0.720	0.585	**0.849**		
PLAT	0.717	0.590	0.345	**0.847**	
SAT	0.611	0.490	0.391	0.719	**0.782**

The items on the diagonal on bold represent the square roots of the AVE; off-diagonalelements are the correlation estimates.

### Structural model fit report

To evaluate the fit of the measurement model, researchers Kline (2011) and Schumacker and Lomax (2010) recommend presenting multiple fit indices to assess the model’s fit. As shown in the Table 6, the study by Jackson et al. (2009) identified the nine most widely used fit indices in SSCI journals, the critical thresholds for a good fit are as follows: the ratio of χ^2^ to degrees of freedom (χ^2^/DF) should fall between 1 and 3, the RMSEA and SRMR values should be less than 0.08, and the TLI, CFI, GFI, and AGFI values should be greater than 0.90. In this study, the measurement model fit indices were as follows: χ^2^/DF = 2.518, RMSEA = 0.068, SRMR = 0.068, TLI = 0.912, CFI = 0.922, GFI = 0.912, and AGFI = 0.900. These results indicate a good fit for the model.

**Table 6:** Satorra-Bentler scaled chi-square adjusted model fitness.

Model fit	Criteria	Model fit of research model
Satorra-Bentlerx^2^	The small the better	513.738
DF	The large the better	204.000
NormedChi-sqr (x2/DF)	1 < x2/DF < 3	2.518
RMSEA	<0.08	0.068
SRMR	<0.08	0.068
TLI (NNFI)	>0.9	0.912
CFI	>0.9	0.922
GFI	>0.9	0.912
AGFI	>0.9	0.9
Scaling correction factor	>1	1.412

**Figure 2:**
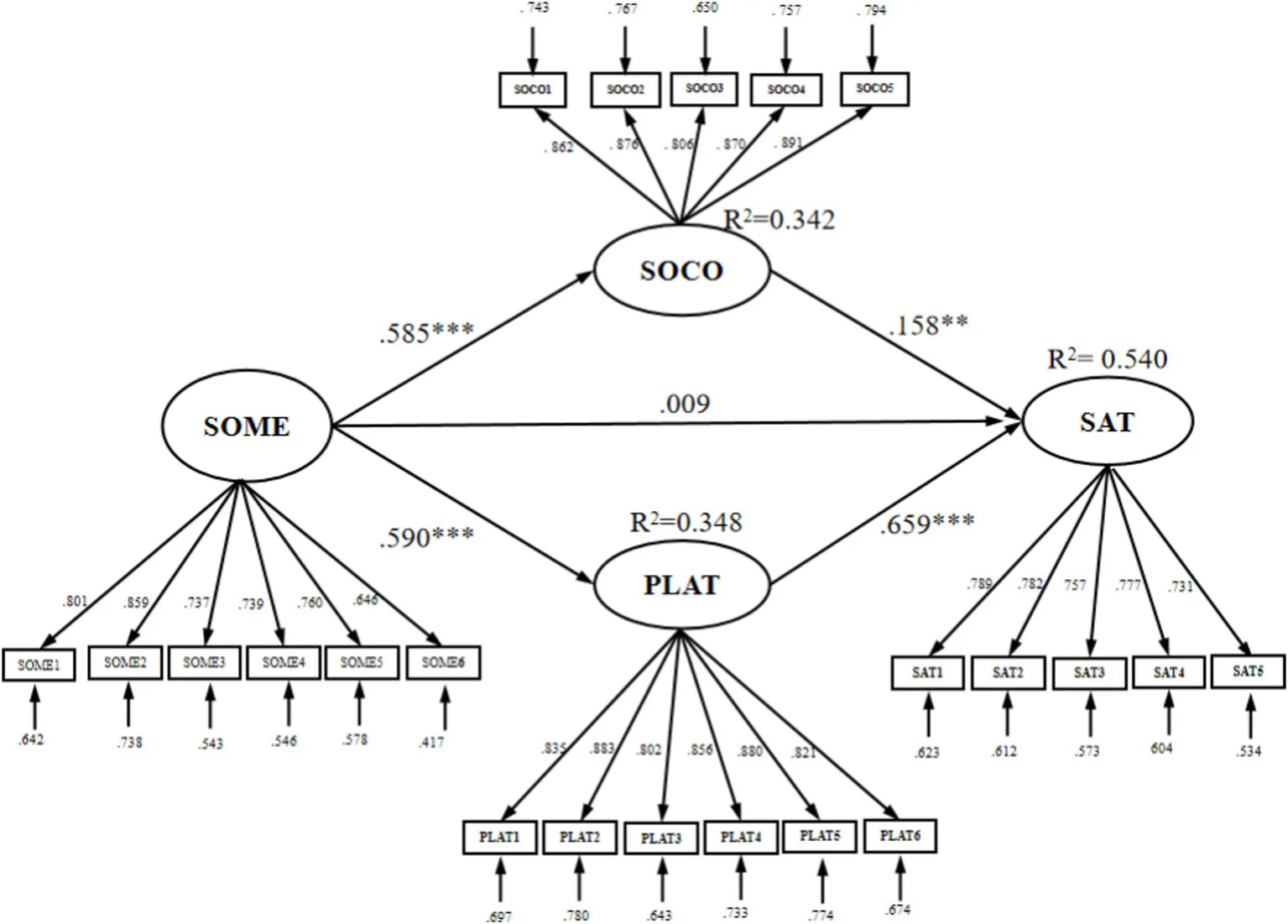
Research statistical model.

The results of this study as shown in Figure 2 largely support the research questions of the model. The explanatory power of Social Media Use (SOME) for Social Connection (SOCO) is 34.2%. The explanatory power of Social Media Use (SOME) for Place Attachment (PLAT) is 34.8%. The explanatory power of Social Media Use (SOME), Social Connection (SOCO), and Place Attachment (PLAT) for Life Satisfaction (SAT) is 54%.

### Mediation effect test

This study employs the Bootstrap resampling method with 5,000 iterations to test the mediation effects. As shown in the analysis Tables 7, 8 of indirect effects for the mediation model below, the total effect of Social Media Use (SOME) → Life Satisfaction (SAT) is significant with *p* < 0.05, and the confidence interval does not include zero [0.218 to 0.431], indicating that the total effect is valid. In the specific indirect effect of Social Media Use (SOME) → Social Connection (SOCO) → Life Satisfaction (SAT), the confidence interval does not include zero [0.005 to 0.125], indicating that the specific indirect effect is valid. This supports the mediation effect H6, suggesting that elderly migrants’ social media use influences their life satisfaction through the level of social connection. Similarly, in the specific indirect effect of Social Media Use (SOME) → Place Attachment (PLAT) → Life Satisfaction (SAT), p < 0.05, and the confidence interval does not include zero [0.187 to 0.334], indicating that this specific indirect effect is also valid. This supports the mediation effect H7, suggesting that elderly migrants’ social media use can also influence their life satisfaction through place attachment. In conclusion, this model demonstrates complete mediation.

**Table 7:** Structural equation model estimation results.

Hypothesis	Structural model path	Unstd. coeff.	Std. coeff.	S.E.	Unstd./S.E.
H1	SOME→SOCO	0.508	0.585	0.052	9.840***
H2	SOME→SAT	0.006	0.009	0.051	0.141
H3	SOME→PLAT	0.499	0.590	0.043	9.865***
H4	SOCO→SAT	0.117	0.158	0.044	2.656**
H5	PLAT→SAT	0.501	0.659	0.052	9.533***

* indicates *p* < 0.05, ** indicates *p* < 0.01, *** indicates *p* < 0.001.

**Table 8:** Analysis of indirect effects for the mediation model.

Effect	Point estimate	Product of coefficients			Bootstrap 5000 times	
					Bias-corrected 95%	
		S.E.	Z-value	*p*-value	Lower bound	Upper bound
Total effect						
SOME→SAT	0.316	0.055	5.739	0.000	0.218	0.431
Specific indirect effect						
SOME→SOCO→SAT	0.060	0.030	1.956	0.050	0.005	0.125
SOME→PLAT→SAT	0.250	0.036	6.928	0.000	0.187	0.334
Direct effect						
SOME→SAT	0.006	0.052	0.116	0.908	−0.097	0.103

## Conclusion and recommendations

This study focuses on the social media use of elderly migrants. Based on a literature review, an integrated model was constructed from the theoretical dimensions of social connection and place attachment to explain and predict whether social media use can enhance elderly migrants’ connection to society, strengthen their sense of place attachment, and ultimately improve their life satisfaction in new environments.

### Conclusion

The model constructed in this study, which uses social connection and place attachment as mediating variables, provides strong explanatory and predictive power for the relationship between social media use and life satisfaction among elderly migrants. However, in terms of direct effects, social media use does not have a significant positive impact on the life satisfaction of elderly migrants. The main conclusions are as follows:

H1: The hypothesis that social media use has a significant positive effect on social connection is supported. This is consistent with the findings of Wu et al. It indicates that social media use affects social connection, validating that elderly migrants can utilize social media in their daily lives to access external societal information in unfamiliar environments, thereby establishing connections with society.

H2: The hypothesis that social media use has a significant positive effect on life satisfaction is not supported. This result differs from the findings of Chen et al. The reasons for this discrepancy can be analyzed from several perspectives:

First, regarding the reasons for migration, caregiving for grandchildren and work are the primary drivers of mobility among elderly migrants. Compared to other older adults, elderly migrants have less leisure time due to their substantial responsibilities, leading to less time for online activities. This limits the relationship between social media use and life satisfaction.

Second, among the 328 survey respondents, the educational level of elderly migrants was generally low. Nearly 30% (29.57%) of respondents had only a primary school education, and over half (53.96%) had less than a middle school education. The limited educational level creates a contradiction with the technical and open nature of social media. On the one hand, elderly migrants face technical barriers when using social media. Without intergenerational support or digital assistance, many elderly people feel overwhelmed by the complexity of social media operations, leading to frustration instead of satisfaction. This frustration can further contribute to feelings of inferiority or helplessness, negatively impacting their life satisfaction. On the other hand, the openness of social media exposes elderly migrants to an overwhelming amount of information, and their limited ability to filter, evaluate, and use this information effectively for social activities leaves them vulnerable. Algorithmic recommendation systems may even lead them to low-quality or false information, resulting in feelings of doubt or insecurity.

Third, the social media use of elderly migrants is largely confined to platforms like WeChat and TikTok. Most of their usage involves group chats, liking posts in Moments, participating in WeChat fitness rankings, or passively browsing content recommended by TikTok. However, social media use encompasses much more, such as content creation, knowledge sharing, and online shopping. The limited functionality of their social media use explains why it does not directly influence their life satisfaction.

Additionally, many factors influence life satisfaction. For elderly migrants, factors like children’s care, grandchildren’s health, adaptation to new environments, and social support play more significant roles than social media use in determining their life satisfaction.

H3: The hypothesis that social media use has a significant positive effect on place attachment is supported. This aligns with the findings of Wang Fang, Xue Tao, and others, confirming that elderly migrants enhance their sense of identification with and dependence on their new living environments through social media use, such as chatting in WeChat groups or watching local TikTok videos.

H4: The hypothesis that social connection has a significant positive effect on life satisfaction is supported. This is consistent with the findings of Ryan D. Duffy and Lai Hongji. It validates that, although elderly migrants live in unfamiliar environments and are prone to loneliness, isolation, and anxiety, staying informed about social affairs and establishing connections with society can reduce negative emotions and improve life satisfaction.

H5: The hypothesis that place attachment has a significant positive effect on life satisfaction is supported. This aligns with the findings of Beru et al., confirming that if elderly migrants develop a sense of identification with and dependence on their new living environment, they are more likely to be satisfied with their migrant lifestyle.

H6: The hypothesis that social connection has a positive mediating effect between social media use and life satisfaction is supported.

H7: The hypothesis that place attachment has a positive mediating effect between social media use and life satisfaction is supported. This suggests that social connection and place attachment, fostered through social media use, can help elderly migrants improve their life satisfaction.

In conclusion, based on the analysis of the unsupported H2 hypothesis (that social media use has a direct significant positive effect on life satisfaction), the findings suggest that enhancing life satisfaction among elderly migrants is feasible by using social media to strengthen their social connection and sense of place attachment.

### Recommendations

Based on the conclusions above, to better meet the psychological needs of elderly migrants, ensure their mental health, enhance their life satisfaction, and foster an environment conducive to active aging, the following recommendations are proposed: First, elderly migrants are encouraged to explore various functions of social media to enrich and simplify their daily lives, thereby improving their life satisfaction. Second, younger generations should place greater emphasis on providing digital support to their elderly relatives who migrate with them. Assisting elderly migrants in using social media in a healthy and scientific manner can help them build social connections and develop a sense of place attachment, ultimately enhancing their life satisfaction. Third, social media platforms should utilize big data to design features and products tailored to the browsing and usage habits of elderly migrants. Enhancing the user-friendliness and age-appropriateness of these platforms can help elderly migrants adapt more quickly and effectively to new living environments. Lastly, governments, communities, and social organizations are encouraged to organize targeted online and offline interaction activities for elderly migrants. These activities can support the formation of social connections and place attachment, contributing to a multifaceted improvement in their life satisfaction.

The present study has several limitations. First, due to the unique characteristics of elderly migrants, including their advanced age and mobility, many factors may influence their life satisfaction. This study focused only on social connection and place attachment. Future research could explore additional factors that may affect the life satisfaction of elderly migrants. Second, although procedural safeguards, including anonymous participation and the use of well-established measurement scales, were implemented to minimize the risk of common method bias, this concern cannot be completely eliminated (Podsakoff et al., 2003, 2012). Future studies may employ longitudinal designs, multi-source data collection, or objective behavioral indicators to further reduce potential method bias. Third, the findings of this study should be interpreted within the context of elderly migrants who use social media. Because the research focuses on the relationship between social media use and life satisfaction, participants who did not use social media were excluded from the final sample. Consequently, the findings may not be fully generalizable to elderly migrants who do not use social media. In addition, the relatively short data collection period and the involvement of undergraduate students in assisting respondents with questionnaire completion may have introduced potential response bias. Future studies are encouraged to employ more diverse sampling strategies to enhance the representativeness of the sample. Finally, future research could combine quantitative and qualitative approaches to gain a deeper understanding of the mechanisms through which social media use influences the life satisfaction of elderly migrants.

## Data Availability

The raw data supporting the conclusions of this article will be made available by the authors, without undue reservation.
